# Determinants of health poverty vulnerability of rural households in Jiangxi Province, China: an analysis based on risk theory

**DOI:** 10.1186/s12889-025-25485-z

**Published:** 2025-12-24

**Authors:** Guoqiu Liu, Zhenzhen Wei, Chunmei Wu, Xueyan Guo, Ming Hao, Xuan Wan

**Affiliations:** School of Public Health and Health Management, Gannan Medical University, Ganzhou, China

**Keywords:** Health poverty vulnerability, Health shocks, Risk response, Risk recovery, China

## Abstract

**Background:**

Although China has made significant progress on poverty alleviation over the past decade, health-related poverty remains an urgent issue in the rural areas of less developed regions, such as central and western China. On the basis of risk theory, this study investigated the determinants of health poverty vulnerability of rural households lifted out of poverty in central China.

**Methods:**

First-hand survey data collected from a convenience sample of 255 previously impoverished households in rural areas of Jiangxi Province in central China in 2024 were used in this study. In this study, an expected poverty vulnerability approach was used to measure health poverty vulnerability, and a three-stage feasible generalized least squares method was adopted to construct the health poverty vulnerability index. A Tobit regression model regression model was used to analyze the influencing factors of health poverty vulnerability.

**Results:**

Among the 255 out-of-poverty households surveyed, 21.18% were found to be vulnerable to health poverty. Our findings indicated that catastrophic health expenditures and labor loss were positively associated with increased vulnerability at the individual and household levels. In contrast, effective chronic disease management appeared to be correlated with reduced health vulnerability, possibly reflecting the supportive role of established social security mechanisms. Regarding health risk response capacity, asset accumulation and social capital may serve as important buffers, though their protective associations were context-dependent. Notably, enrollment in basic health insurance was linked to higher health-related poverty vulnerability, suggesting limited effectiveness in mitigating vulnerability among low-income groups. From a risk resilience perspective, higher educational attainment and a greater proportion of working-age family members were associated with lower vulnerability, though underlying mechanisms warranted further investigation.

**Conclusions:**

Rural households in Jiangxi Province, China has remained susceptible to returning to poverty due to health shocks. These results underscore the need for more human capital investments to enhance the capacity of rural households to withstand health shocks, and a resilient health security system that accurately identifies vulnerability pathways and integrates strengthened social protection, asset-building initiatives.

## Introduction

Poverty, as one of the social determinants of health, has a bidirectional dynamic relationship with population health outcomes. Research in health economics demonstrates that health deprivation is not merely a consequence of poverty but that it also perpetuates a vicious cycle of "poverty-induced illness and illness-induced poverty" through mechanisms such as human capital depreciation and catastrophic health expenditures [49, 56, 64]. Joint monitoring by the World Health Organization and the World Bank has revealed that approximately 344 million individuals globally faced extreme poverty due to healthcare costs in 2019, with low-income countries experiencing particularly high catastrophic health expenditure rates of 19.8% [65]. In the Chinese context, the concept of poverty usually refers to the living state of lacking sufficient income to meet a family’s basic needs. Although China has successfully eliminated absolute poverty by 2020 [52], it continues to confront health-related poverty vulnerabilities among those lifted out of poverty. According to official standards, a household with per capita annual income less than 2300 RMB was regarded as living in absolute poverty in rural China [52].

Empirical studies has indicated that, of the poor rural households, 10 million were impoverished due to illness, accounting for 40% of all poor households [72]. In central and western China, constrained healthcare access, limited income-generating capacity, and lower levels of educational attainment increases the likelihood of local individuals or families falling into poverty due to health-related problems [9, 69, 72]. This paradox underscores that absolute poverty primarily focuses on economic deprivation metrics, whereas systemic deficiencies in healthcare accessibility, essential service quality, and health literacy levels still persist in rural China. Consequently, developing a health-related poverty-vulnerability assessment framework has emerged as a critical tool for identifying critical thresholds of return-to-poverty risk and disrupting the health-poverty nexus. This methodological innovation enables targeted interventions to strengthen health system resilience and sustain poverty alleviation achievements in post-2020 China.

The concept of poverty vulnerability was first proposed by the [63]. Poverty vulnerability was defined as the likelihood of a family falling into poverty in the future [63]. Health poverty vulnerability refers to the probability of falling into poverty in the future due to health-related problems [9, 69, 72]. Health poverty vulnerability, as a novel framework in poverty research from a social risk perspective, transcends traditional static conceptualizations of poverty. Its core premise lies in prospectively assessing the risk of individuals or households falling into persistent poverty due to insufficient risk-coping capabilities when facing health status abnormalities caused by sudden factors such as diseases, accidents, or environmental changes [55].

Health shocks are typically defined as unforeseen and uncontrollable exogenous events, including acute illnesses, accidental injuries, or newly diagnosed chronic diseases [19]. Existing research suggests that the economic impacts of health shocks on households manifest in both abrupt and cumulative forms. Acute shocks often impose an immediate financial burden through sharp increases in out-of-pocket medical expenditures, whereas chronic or recurrent health problems gradually erode household income bases via long-term declines in labor capacity and the loss of employment opportunities [57]. As a core driver of health-poverty vulnerability, their impacts follow an "exposure-sensitivity" framework. With respect to risk exposure, most rural Chinese households face considerable health shocks due to deficiencies in healthcare accessibility and service quality [22]. In terms of shock sensitivity, health risk vulnerability manifests through labor capacity erosion. Sen’s capability poverty theory posits that severe illness-induced labor capability deprivation may trigger "capability traps," creating a vicious cycle between health deficits and poverty [49]. A longitudinal study in rural China corroborates this finding, demonstrating that each additional care-dependent member reduces household labor participation rates by 53% [56].

To address these challenges, academic discourse distinguishes between risk response capacity and risk recovery capacity. The former concept builds on Townsend's coping strategies theory of poverty, emphasizing asset liquidation and social capital mobilization as buffers against medical expenditures [53]. As a core buffer mechanism in poverty vulnerability governance systems, this capacity inherently involves resource reallocation to preempt health-related risks, which are constrained jointly by resource endowments and institutional environments. In terms of resource endowments, critical dimensions include material assets, human capital, and social capital. Resource inadequacy among households that have been recently lifted out of poverty exacerbates their health-poverty vulnerability. Empirical studies on post-poverty rural Chinese households reveal that when savings fall below critical thresholds and out-of-pocket medical expenses rise, recidivism risks escalate [66], corroborating Sen's thesis on "resource deprivation leading to capability traps." Institutional constraints amplify coping capacity failures through two pathways: weakened risk-sharing efficacy and imbalanced social support systems.

Health risk resilience originates from ecological resilience theory [24]. After adaptation in social science disciplines, it has been further applied to household economics, referring to a household’s capacity to gradually restore livelihoods by adjusting labor allocation patterns and rebuilding resource foundations following adverse health shocks [67].This theoretical framework innovatively integrates Sen's capability approach with Grossman's health capital theory [21, 49], elucidating the dual nature of health resources in post-poverty households: health functions both as a core economic productivity factor and as a "safety reserve" against health shocks. However, in rural areas with insufficient primary healthcare coverage, sudden health crises often trap households in a vicious cycle of "medical debt-labor capacity erosion-income deterioration," ultimately reversing poverty alleviation achievements [61, 73]. These findings underscore rural households' constrained capacity to mitigate health risks due to socioeconomic disadvantages and limited health management capabilities, thus positioning health-related poverty vulnerability assessment as critical for preventing poverty recurrence.

On the basis of a profound understanding of household behavior and vulnerability under health shocks, academia has begun to systematically incorporate risk theory into the framework of health-related poverty vulnerability.

The concept of risk, initially rooted in medieval notions of “fate” and “adventure,” gradually evolved with the expansion of commerce into a rational framework for assessing uncertainty [47]. From the mid-twentieth century onward, recurrent crises such as environmental pollution and nuclear accidents underscored the inherent unpredictability and wide diffusion of modern risks. In Risk Society, Ulrich Beck introduced the concept of “manufactured risks” [3], highlighting that risks emerge from the internal dynamics of modern institutional systems and disproportionately burden disadvantaged groups by exacerbating structural vulnerabilities. By the 1990 s, poverty research began to transcend income-centric approaches and turned to household vulnerability in the face of uncertain shocks. Within this paradigm, the World Bank’s World Development Report 2000/2001 incorporated the “health risk–poverty trap” into the poverty vulnerability framework [41, 63], emphasizing that sudden and uncertain health shocks can undermine household livelihoods and perpetuate cycles of health-induced poverty.

Building upon this foundation, Alwang and colleagues achieved a conceptual breakthrough with their tripartite vulnerability analysis model [1]. This framework operationalizes three analytical dimensions: risk exposure through the quantification of health shock probabilities, sensitivity as measured by economic system fragility, and adaptive capacity incorporating material resource buffers and social capital reserves.

However, a key limitation of this tripartite vulnerability analysis model was its predominant focus on identifying exposure and sensitivity, neglecting the critical interactions between socioeconomic factors and health shocks. Consequently, it offered an incomplete explanation of the generative mechanisms underlying health shocks induced vulnerability and insufficiently elucidated post-shock recovery trajectories. Subsequent theoretical advancements have integrated capability poverty theory to explain how health shocks exacerbate vulnerability through human capital depreciation [49], whereas health capital theory has quantified the erosive effects of disease burdens on household reproductive capacity [21]. These theoretical synergies were the catalyst for the development of a dynamic "health shock‒response‒recovery" model, which fundamentally reshaped the understanding of health-related poverty mechanisms. The evolution of the framework reflects the progressive recognition of health shocks as both economic destabilizers and systemic vulnerabilities requiring multidimensional interventions.

While the predictive value of health-related poverty vulnerability is widely acknowledged, current scholarship exhibits notable limitations. Most studies adopt static perspectives that focus on health status, medical services, insurance coverage, and living environments [12, 26, 32, 33, 46, 48], while neglecting dynamic risk mechanisms. Although some scholars have examined health shocks and social capital impacts [51, 58, 62, 77, 79], comprehensive analyses that integrate health shocks, risk response and risk recovery remain scarce. In recent years, a growing body of research has also turned to the health effects of environmental policies [15, 30]. For instance, evidence shows that clean heating initiatives can significantly improve individual health outcomes [30]. Nevertheless, these studies largely remain confined to the health outcome dimension and have yet to incorporate such findings into the dynamic analytical framework of poverty vulnerability, thereby limiting deeper insights into the mechanisms linking environmental policy, health outcomes, and vulnerability evolution. Methodological inconsistencies on vulnerability indices further hinder cross-study comparability and policy applicability [29, 33, 43–45]. Crucially, insufficient engagement with risk theory limits the understanding of temporal dynamics in health‒economic interactions [16, 39, 59, 76].

To address these gaps, this study constructs a health-related poverty vulnerability framework through a risk lens, incorporating three dimensions: health shocks, risk response capacity, and risk recovery capacity. By measuring the level of health poverty vulnerability and identifying its determinants, this research aims to inform targeted interventions and optimize health policies in the post-poverty relief era and provide both theoretical and practical insights for sustaining poverty alleviation achievements in less developed areas.

## Materials and methods

### Data sources

The survey was conducted in the rural areas of Ganzhou, Jiujiang, and Yichun, three prefecture-level cities in Jiangxi Province in the southern central part of China. In 2015, the Chinese central government issued a policy document on winning the battle against poverty, ensuring that the rural poor would be lifted out of poverty by 2020 [5]. At the beginning of 2015, Jiangxi Province reported 2.76 million impoverished residents, with a rural poverty incidence rate of 5.8% [27], which is slightly higher than the national average rate of 5.7% [42]. The cities of Ganzhou, Jiujiang, and Yichun are located in southern, northern, and western Jiangxi Province, respectively. The combined territory accounts for approximately 46% of the province’s total area, with rural populations constituting 44.6% of Jiangxi’s rural residents [27]. Ganzhou, as the largest prefecture-level city in Jiangxi in terms of both area and population, had 1.1433 million poverty-stricken residents in 2014, which represented 40% of the province’s total impoverished population, thus making its poverty alleviation outcomes pivotal to the province’s overall anti-poverty campaign [2]. Jiujiang, situated at the confluence of the Yangtze River and Poyang Lake, faces heightened ecological fragility-driven poverty recurrence risks due to frequent flood disasters. Yichun, a traditional agricultural region, presents increased livelihood vulnerability among poverty-alleviating households due to its mono-industrial economic structure. These three cities demonstrate strong representativeness in terms of post-poverty alleviation population scales and risk factors for returning to poverty, providing a robust geographical sample for studying health-related poverty vulnerability mechanisms in transitional households.

On the basis of the aforementioned context, a convenience sampling method was adopted, and rural areas of Ganzhou, Jiujiang, and Yichun in Jiangxi Province were selected as the survey sites. In each city, counties ranking in the top two in terms of the number of poverty-alleviated households were first identified, and then 2–3 townships under their jurisdiction were selected as the sampling frame, taking into account population size, geographic location, and transportation accessibility. Fourteen student volunteers from Gannan Medical University, who returned home during their vacation, conducted household surveys in their own villages and neighboring communities. According to the criterion of “no fewer than 10 households per village,”, they conducted face-to-face questionnaire interviews with registered poverty-alleviated households. Upon entering each village, investigators first obtained a list of poverty-alleviated households from the village committee and then conveniently selected respondents on site based on actual circumstances. All volunteers underwent uniform training prior to fieldwork to standardize procedures for obtaining informed consent, safeguarding participant confidentiality, and ensuring data quality. Among the 300 questionnaires distributed, 270 were successfully obtained, and 255 were valid, yielding a response rate of 90.00% and a validity rate of 85.00%.

### Variables and definitions

#### Health shocks

This study was primarily interested in the intensity of health shocks. Health shock intensity was measured through three indicators: the health status of household members, the caregiving burden (including whether there are chronic disease patients in the family, whether there are family members needing long-term care, and the burden of medical expenses), and the incidence of catastrophic health expenditures. This framework enables systematic analysis of how health shocks influence post-poverty financial stability and social welfare outcomes among rural households.

Self-rated health, as a key subjective indicator of health measurement, reflects individuals' comprehensive subjective evaluation of their own health status, functional capacity, and life adaptability. It effectively reveals individuals' self-perception and assessment when facing health risks and economic pressures [54]. In this study, a self-rated health scale is employed for measurement, and the respondents' health evaluation data are collected using a five-point Likert scale (excellent, good, fair, poor, and very poor). The validity of this discrete variable measurement has been verified by domestic and international studies [10, 23].

Family care burden refers to the multidimensional pressure accumulation caused by chronic illness or disability among family members, manifested through persistent labor resource depletion, economic resource encroachment, and escalating psychological stress [36]. According to Sen's capability poverty theory, this concept represents the persistent deprivation of family labor capacity due to health issues [49]. The study evaluated family care burden through three dimensions: chronic disease prevalence, long-term care needs, and medical expenditure burden. For chronic disease prevalence, a binary variable that measures the presence of chronic disease patients diagnosed by medical institutions within the family is applied. Long-term care needs are identified through the objective fact of "whether family members require continuous life care for over six months due to disability or dementia" [74].

Medical expenditure burden is measured through the subjective perception question "Does family medical support cause pressure on daily life?" This measurement system forms an objective evaluation framework distinct from the subjective dimension of self-rated health, utilizing three indicators: chronic disease existence, long-term care necessity, and medical burden perception. The validity of this instrument is supported by Wagstaff et al.'s longitudinal study in rural China, which demonstrates that each additional care-dependent family member leads to a 53% cliff-like decline in labor participation rates [56], empirically corroborating Sen's health-related capability deprivation theory [49].

Catastrophic health expenditure refers to uncertain health expenditures that households or individuals may incur when facing health shocks that potentially cause significant economic impacts. These expenditures carry uncertainty and potential risks that may drive families back into poverty or exacerbate existing poverty conditions. Mechanistically, this concept encompasses both direct medical expenditures and indirect income losses from health-related human capital depreciation. With respect to quantification standards, some studies propose the use of 40% of household health expenditures in proportion to disposable income as the benchmark for catastrophic health expenditures [68]. However, given the unique consumption structure of rural Chinese households, threshold adaptation is required [22, 66]. This study combines sample family consumption elasticity analysis to set 50% of the medical expenditure proportion to total consumption expenditure as the identification threshold for catastrophic health expenditure.

#### Health risk response capacity

This study operationalizes health risk coping capacity using the following indicators: household assets (including income, real estate, and vehicles), health insurance coverage (encompassing basic public insurance, commercial insurance, and uninsured household members), and social capital (reflected in the availability of long-term care from relatives/friends and access to financial support from kinship networks). This multidimensional framework systematically captures a household’s ability to buffer health shocks while reflecting structural limitations in rural institutional contexts.

Within the dimension of household characteristics, family economic capital and material assets constitute the core resource endowment base for resisting health shocks. According to Townsend's theory of household coping strategies, household income levels directly determine their buffer resilience in managing health-related expenditures, whereas fixed assets such as real estate and vehicles serve as reserve material resources for risk mitigation [53]. Consequently, greater capacity to generate income and accumulate assets enhances a household's ability to withstand health-related shocks. Notably, rural Chinese households predominantly adopt labor-intensive income-generating models, where family size is significantly positively correlated with labor supply capacity, thereby influencing income levels [54]. This structural characteristic underscores the close relationship between household size and health poverty vulnerability. The study employs questionnaire data on annual household disposable income (including wage income, business income, and transfer payments), real estate holdings, vehicle ownership, and the registered household population to assess household characteristics. Health insurance serves as a critical tool for managing health risks and mitigating disease shocks, particularly demonstrating significant poverty alleviation effects for health-compromised populations. Basic medical insurance plays a central role in reducing enrollees' healthcare burdens, whereas commercial insurance supplements basic coverage by addressing higher-tier medical security needs. This study evaluates health insurance participation through binary variables, i.e., enrollment in basic medical insurance and enrollment in commercial medical insurance, and quantifies the number of uninsured family members. Social capital represents a vital dimension of resource acquisition within social network structures, fundamentally enabling access to financial and human capital through interpersonal relationships [4]. Granovetter's "strength of ties" theoretical framework evaluates social capital in four dimensions: interaction frequency, emotional depth, intimacy level, and degree of reciprocity, with strong-tie social capital facilitating higher-density resource exchanges [20]. This perspective holds particular relevance for understanding social capital functions in rural Chinese contexts characterized by inadequate formal institutional safeguards. The relationship networks formed through blood ties and geographical proximity emerge as critical factors enabling rural households to counter health risk shocks through informal mechanisms such as long-term care support and economic assistance. Guided by this theory, the study measures social capital strength through indicators assessing the availability of long-term care from relatives/friends and access to economic support networks.

#### Health risk resilience

This study operationalizes health risk resilience through three indicators: age, educational attainment, and the number of working-age household members.

Age, as a life-cycle indicator [28], reveals intergenerational differences in health shocks through interactions between individual physiological characteristics and family labor force structures. This study categorizes family members into five age groups on the basis of chronological age, physiological capacity, and China’s labor market dynamics: the nonlabor dependency period (0–19 years), the core labor force active period (20–39 years), the occupational stability challenge period (40–59 years), the retirement transition period (60–79 years), and the advanced health-sensitive period (80 + years). This classification elucidates differential recovery capacities across age cohorts when confronting health shocks. Family educational attainment, a critical moderating variable for health poverty vulnerability, enhances health risk resilience through knowledge accumulation and health information processing capabilities [38]. The study evaluated these competencies using educational tiers: illiterate/semiliterate, primary school, junior high school, and senior high school or above. Research has demonstrated that highly educated households exhibit greater wealth acquisition capacity, medical knowledge retention, and health management practices than do less educated households [34, 54]. In the labor structure dimension, the family labor force proportion quantifies the percentage of healthy working-age members (16–60 years) within registered household populations, reflecting the labor reserve capacity to buffer health shocks. This mechanism operates through intrahousehold labor reallocation to compensate for workforce attrition caused by health crises [24]. By integrating the metrics of physiological capital, human capital accumulation, and labor structure flexibility, these indicators collectively construct a measurement framework for health risk resilience capacity.

### Statistical analysis

Under the risk theory framework, in this study, a health poverty vulnerability index, consisting of three dimensions—health shocks, risk response, and risk resilience—is constructed. Regarding the measurement of health poverty vulnerability, the internationally recognized approaches mainly include the vulnerability as expected poverty (VEP) [6], the vulnerability as expected utility (VEU) [31], and the vulnerability as low expected utility from risk exposure (VER) [8]. The VEU approach assesses poverty vulnerability by calculating the gap between the expected utility at the poverty line and the expected utility of household consumption. However, it relies on a single utility function, which may fail to capture household heterogeneity in preferences, and it requires data with considerable temporal coverage, thereby limiting its practical applicability. The VER approach estimates poverty vulnerability by evaluating the sensitivity of household welfare to risk shocks. While it highlights households’ coping capacity against realized risks, it lacks forward-looking characteristics. The VEP, proposed by Chaudhuri and colleagues, estimates health-related poverty vulnerability by predicting future household per capita consumption through observable variables and shocks [6]. This framework assumes that expected household per capita consumption follows a log-normal distribution and calculates the probability of consumption falling below a specific threshold to derive the vulnerability index [40, 61, 80]. Grounded in the dynamic characteristics of health‒poverty vulnerability, VEP theory aims to quantify welfare losses caused by adverse health conditions among household members and assess future poverty risks. This method not only evaluates health-poverty vulnerability but also addresses the limitations of panel data by enabling vulnerability estimation using cross-sectional data.

When applying the VEP approach to calculate health poverty vulnerability, it is first necessary to define the poverty line. Current practices among Chinese scholars predominantly adopt international poverty lines of 2 or 3 US dollars per day. This study employs the poverty line of 2 US dollars per day to compute the health-poverty vulnerability index. We employ Chaudhuri and colleagues’ three-stage feasible generalized least squares (FGLS) method to obtain auxiliary estimates of the VEP model. FGLS iteratively estimates the variance and covariance structure of the error terms to correct for heteroskedasticity and error correlation, thereby improving the robustness of parameter estimation and the reliability of the model. This approach is particularly effective in enhancing model performance when dealing with complex data such as health-related poverty vulnerability. The specific procedures are as follows:

First, we estimate the household consumption equation, and use the squared residuals obtained from the regression as an indicator of consumption volatility for OLS estimation. The estimation equation is as follows:

1 $$\mathrm{ln}\:{C}_{h}={X}_{h}+{e}_{h}$$

2 $$\widehat e_h^2=X_h\rho+\varepsilon_h$$

Here, $\text{ln}{C}_{h}$ represents the natural logarithm of household consumption, reflecting variations in household consumption levels; ${X}_{h}$ denotes a set of observable variables influencing per capita consumption, including health-related poverty vulnerability factors and measures the intensity of consumption volatility. Drawing on risk theory, capability poverty theory, sustainable livelihoods theory, and poverty dynamics theory, in this study, a health‒poverty vulnerability index, consisting of three dimensions*—* health shocks, risk response capacity, and risk resilience*—*is constructed.

Second, using the fitted values obtained in the first step, we conduct a weighted regression of the logarithm of household consumption and the logarithm of the squared residuals to obtain the FGLS estimates. This allows us to estimate future consumption volatility as well as the expected value of the logarithm of consumption, as shown in Eqs. (3) and (4):

3 $$\widehat{\mathrm E}(\ln\;C_{\mathrm h}\vert{\mathrm X}_{\mathrm h})={\mathrm X}_{\mathrm h}{\widehat{\mathrm\beta}}_{\mathrm{FGLS}}$$

4 $$\widehat{\mathrm V}(\ln\;C_{\mathrm h}\vert{\mathrm X}_{\mathrm h})=\widehat\sigma_{\mathrm e,\mathrm h}^2={\mathrm X}_{\mathrm h}{\widehat{\mathrm\rho}}_{\mathrm{FGLS}}$$

Finally, assuming that the future consumption level of the study population follows a log-normal distribution, the health poverty vulnerability index can be expressed as:

5 $$V_ul_h=Prob\left(\ln\;C_h<\ln Z\vert X_h\right)=\varnothing\left(\frac{\ln Z-\ln X_h{\widehat\beta}_{FGLS}}{\sqrt{X_h{\widehat\rho}_{FGLS}}}\right)$$

where denotes the logarithmic value of the poverty line. In this study, we follow the internationally recognized standard and adopt the 2 US dollars per day threshold to calculate the health poverty vulnerability index. The vulnerability line reflects the probability that the study population will fall into poverty in the future. The vulnerability line reflects the probability of the sampled households falling into poverty in the future. Zhang and Wan [78] found that the precision of prediction varies depending on the vulnerability line, and that setting the line at 0.5 can improve predictive power. Consequently, households with a probability exceeding 50% are classified as health-poverty vulnerable. The study further employs a Tobit regression model to analyze contributing factors, specifying health-poverty vulnerability as the dependent variable and health shocks, risk response, and risk resilience as independent variables, with gender and marital status included as control variables. This framework quantifies the relative contributions of each determinant to health-poverty vulnerability. All statistical analyses are conducted using IBM SPSS 27.0 software. A *P* value less than 0.05 was considered statistically significant.

## Results

### Sample characteristics

This study administered household questionnaires to 300 poverty-alleviating households in Ganzhou, Jiujiang, and Yichun, Jiangxi Province, and collected 255 valid responses, with a 94.44% valid response rate. The key findings include the following: 35.12% (87 respondents) were male and 65.88% (168 respondents) were female, and 24.71% (63 respondents) were unmarried, divorced, or widowed. These statistics represent a gender-marital structure that is consistent with rural labor migration patterns. With respect to health risk shocks, 69.41% (177 respondents) reported self-rated health as fair or better, yet the chronic disease prevalence rate reached 55.68% (142 respondents), with 19.22% (49 respondents) requiring long-term care. Notably, 32.94% (84 respondents) perceived medical expenses as burdensome to daily life, including 10.59% (27 respondents) who experienced catastrophic health expenditures, data which empirically validate the health‒poverty vicious cycle theory. For health risk response capacity, 71.76% (183 respondents) had 46 household members, while 84.31% (215 respondents) reported annual income below 100,000 yuan. Asset deficiencies were observed in 33.73% (86 respondents) had no real estate and 18.43% (47 respondents) had no vehicles, highlighting resource-capacity mismatches in risk buffering. Critical vulnerabilities included 20.78% (53 respondents) without basic medical insurance, 34.12% (87 respondents) lacking access to informal caregiving, and 28.24% (72 respondents) deprived of economic support from social networks. In health recovery capacity metrics, the 20 to 59 age cohort comprised 63.14% (158 respondents), whereas 78.03% (199 respondents) attained primary or junior high school as their highest level of education level. Only 64.27% (164respondents) demonstrated labor force participation rates exceeding 40% among registered members, underscoring constraints in human capital and labor adaptability within post-poverty alleviation households. In terms of health recovery capacity metrics, the 20–59 age cohort comprised 63.14% (158respondents), whereas 78.03% (199respondents) attained primary or junior high school as their highest level of education. Only 64.27% (164 respondents) demonstrated labor force participation rates exceeding 40% among registered members, underscoring constraints in human capital and labor adaptability within post-poverty alleviation households (see Table 1).

**Table 1:** Survey overview (*n* = 255)

	Variable name	Demographic characteristics	*n*	Percentage (%)	Cumulative percentage(%)
Individual attributes	Gender	Male	87	35.12	35.12
		Female	168	65.88	100
	Marital status	Not married/divorced/widowed	63	24.71	24.71
		Married	192	75.29	100
Health shocks	Self-rated health status	Very good	121	47.45	47.45
		Good	39	15.29	62.74
		Fair	17	6.67	69.41
		Poor	54	21.18	90.59
		Very poor	24	9.41	100
	Chronic disease in household	Yes	142	55.69	55.69
		No	113	44.31	100
	Need for caregiving in household	Yes	49	19.22	19.22
		No	206	80.78	100
	Catastrophic health expenditure(%)	0–50	228	89.41	89.41
		> 50	27	10.59	10.59
	Financial burden from medical costs	No burden	171	67.06	67.06
		Manageable burden	12	4.71	71.77
		Significant burden	11	4.31	76.08
		Unaffordable	61	23.92	100
Health risk response capacity	Household size(members)	0–3	25	9.80	9.80
		4–6	183	71.76	81.57
		> 7	47	18.43	100
	Annual household income(CNY)	0–19999	53	20.78	20.78
		20,000–49999	71	27.84	48.62
		50,000–99999	91	35.69	84.31
		> 100,000	40	15.69	100
	Real estate(unites)	0	86	33.73	33.73
		1	164	64.31	98.04
		≥ 2	5	1.96	100
	Vehicle ownership(unites)	0	47	18.43	18.43
		1	159	62.35	80.78
		≥ 2	49	19.22	100
	Basic medical insurance	Yes	202	79.22	79.22
		No	53	20.78	100
	Commercial supplementary medical insurance	Yes	107	41.96	41.96
		No	148	58.04	1
	Access to long-term care from relatives/friends	Yes	168	65.88	65.88
		No	87	34.12	100
	Access to economic support from relatives/friends	Yes	183	71.76	71.76
		No	72	28.24	100
Health risk resilience	Age (years)	0–19	8	3.14	3.14
		20–39	79	30.98	34.12
		40–59	82	32.16	66.27
		60–79	83	32.55	98.82
		80–100	3	1.18	100
	Educational attainment	Illiterate/semi-illiterate	25	9.80	9.80
		Primary school	81	31.76	41.57
		Junior high school	118	46.27	87.84
		Senior high school	31	12.16	100
	Labor force proportion (%)	0–19.99	51	20.00	20.00
		20–39.99	40	15.69	35.69
		40–59.99	42	16.47	52.16
		60–79.99	64	25.10	77.25
		80–100	58	22.75	100

### Health poverty vulnerability distribution and model

Among the 255 out-of-poverty households surveyed, 201 (78.82%) had a poverty vulnerability index of less than 0.5, and those with a poverty vulnerability index equal to or greater than 0.5 accounted for 21.18% of the total number. That is, 21.18% of rural out-of-poverty households are vulnerable to health-related poverty in the future. Table 2 presents the distribution of the health poverty vulnerability index.

**Table 2:** Distribution of health poverty vulnerability

Index value	*n*	Mean	Standard deviation	Vulnerable rate (%)
< 0.5	201	0.07	0.12	78.82
0.5–1	54	0.97	0.10	21.18
Total	255	0.26	0.38	100.00

The health-related poverty vulnerability index ranges from 0 to 1 and represents a typical censored continuous variable. Traditional Ordinary Least Squares (OLS) regression models are inadequate for accurately handling such data characteristics. To estimate the effects of various factors on health-related poverty vulnerability, this study employs a Tobit regression model. A Tobit regression model was set such that when the latent variable y* was less than or equal to 0, the explanatory variable *y* = 0. When y* was greater than 0, the explanatory variable y was equal to y* itself, and it was also assumed that the perturbation term *ui* obeyed a mean of 0 and a variance of the *σ*^2^ normal distribution. Descriptive statistics for the control variables are presented in Table 3. The marital status variable was excluded from the model due to a variance inflation factor (VIF) greater than 10, whereas the remaining variables all have VIFs below 10, indicating that is not a serious concern(see Table 4).

**Table 3:** Descriptive statistics of control variables for the health poverty vulnerability index

Variable name	n	x ± s
Gender		
Male	87	0.08 ± 0.17
Female	168	0.36 ± 0.43

**Table 4:** Results of VIF test

Variable name	VIF	1/VIF
Gender	1.26	0.80
Self-rated health status	2.38	0.42
Chronic disease in household	3.23	0.31
Need for caregiving in household	1.88	0.53
Catastrophic health expenditure	1.85	0.54
Household size	3.64	0.27
Annual household income	3.57	0.28
Real estate	3.53	0.28
Vehicle ownership	1.11	0.90
Basic medical insurance	3.77	0.27
Commercial supplementary medical insurance	2.06	0.48
Access to long-term care from relatives/friends	1.88	0.53
Access to economic support from relatives/friends	2.48	0.40
Age	1.92	0.52
Educational attainment	4.85	0.21
Labor force proportion	7.57	0.13

### Regression analysis

Health shock factors include self-assessed health status, chronic disease patients, family members in need of long-term care, and medical expenditures, of which self-assessed health status (β = 0.02, *P* < 0.001), family members in need of long-term care (β = 0.09, *P* = 0.030), and medical expenditures (risky health expenditures and medical expenses on life) (β = 0.09, *P* = 0.010 and β = 0.04, *P* = 0.010) were positively associated with health poverty vulnerability. Chronic disease patients (β = −0.10, *P* < 0.001) was negatively correlated with health poverty vulnerability (see Table 5). The overall study results indicated that health shock factors were positively associated with health poverty vulnerability.

**Table 5:** Regression analysis on health poverty vulnerability

Independent variables	Regression coefficient	Standard error	95% *CI*	
Self-assessed health status	0.02***	0.01	0.01	0.03
Chronic diseases	−0.10***	0.02	−0.14	−0.06
Members in need of long-term care	0.09*	0.04	0.01	0.18
Risky health expenditure	0.09*	0.03	0.02	0.15
Burden of medical expenses	0.04*	0.01	0.01	0.07
Household size	0.05***	0.01	0.03	0.06
Household Income	−0.06***	0.01	−0.08	−0.04
Property	−0.07*	0.03	−0.13	−0.02
Car	0.00	0.01	−0.02	0.01
Basic medical insurance	0.58***	0.04	0.50	0.66
Supplementary medical insurance	−0.01	0.02	−0.04	0.03
Uninsured	0.04*	0.01	0.01	0.07
Long-term care from friends	−0.01	0.02	−0.04	0.02
Financial support from friends	0.01	0.02	−0.03	0.05
Age	0.02*	0.09	0.00	0.04
Highest educational level	−0.01	0.02	−0.05	0.02
Labor force ratio	−0.18***	0.06	0.07	0.29

^*^P < 0.05, ***P < 0.001

Health risk response capacity factors include household size, household assets (household income, property and car), medical insurance (basic medical insurance and supplementary medical insurance) and social capital. Household size (β = 0.05, *P* < 0.001) and participation in basic medical insurance (β = 0.58, *P* < 0.001) were positively associated with health poverty vulnerability, whereas household assets (β = −0.06, *P* < 0.001 and β = −0.07, *P* = 0.010) were negatively correlated with health poverty vulnerability (see Table 5). Those findings indicated that health risk response capacity factors were negatively associated with health poverty vulnerability.

Health risk resilience factors including age, highest level of education attained and labor force ratio. Although age (β = 0.02, *P* = 0.010) were positively associated with health poverty vulnerability, the labor force ratio (β = −0.18, *P* < 0.001) in the family was negatively associated with health poverty vulnerability (see Table 5). Those findings indicated that health risk resilience factors were negatively associated with health poverty vulnerability.

## Discussion

### Health shock and health poverty vulnerability

#### The impact of self-assessed health status on health poverty vulnerability

The results indicated that self-assessed health status had a positive association with health poverty vulnerability, with a regression coefficient of 0.02 (see Table 5). Contrary to the conventional assumption that better self-assessed health status would be associated with lower vulnerability, our findings indicate that higher self-assessed health levels were associated with higher health poverty vulnerability. This result contrasts with conclusions drawn from previous literature [7]. This phenomenon may reflect that self-rated health, as a subjective measure, involves potential cognitive biases. These biases, intertwined with individuals' subsequent behavioral choices and resource allocation patterns, may collectively correlate with varying degrees of vulnerability [50].

#### The impact of chronic disease patients on health poverty vulnerability

The study indicated that there was a negative association between chronic disease patients and health poverty vulnerability, with a regression coefficient of −0.10 (see Table 5). This suggests that households with chronic disease patients may exhibit a lower risk of falling into poverty compared to those without, a finding consistent with previous research [11, 17]. Field survey observations further indicated that poorer family health status, such as having a member requiring long-term care, was generally associated with a higher degree of health poverty vulnerability among registered poverty-alleviated households. However, when chronic conditions within a family were effectively managed, while there may be implications for individual productivity, no significant association was observed with income-based poverty vulnerability. Furthermore, the implementation of a contracted management service policy for chronic disease families has been associated with a measurable reduction in poverty vulnerability [18].

#### The impact of family members in need of long-term care on health poverty vulnerability

There was a positive association between family members in need of long-term care and health poverty vulnerability, with a regression coefficient of 0.09 (see Table 5). This relationship may involve multiple mechanisms. First, family members in need of long-term care are usually found in special groups, such as the chronically ill, the disabled, and the elderly. Long-term care requires a significant monetary investment from family members, which may lead to an increased financial burden on family members and even financial difficulties, especially for low-income families. Second, it affects the patient's ability and opportunity to engage in productive activities. Furthermore, long-term care requires family members to invest a large amount of time and energy, which reduces the labor time of other family members and may also affect other family members' health status, including physical health and mental health, thereby resulting in further reduced family income [81].

#### The impact of risky health expenditures on health poverty vulnerability

There was a positive association between risky health expenditure and health poverty vulnerability, with a regression coefficient of 0.09 (see Table 5). High health expenditures may have a significant association with a household's financial situation. In particular, for households that have just escaped from poverty, healthcare expenditures may constitute a large proportion of their household income, leading to financial difficulties and increasing their health poverty vulnerability. High medical expenditures reduce patients' chances of recovery, with approximately 50% of patients who should have been hospitalized not being hospitalized for financial reasons [13, 14]. Whether medical expenses are a burden on life has a positive association on health poverty vulnerability, with a regression coefficient of 0.04 (see Table 5), This finding provides further evidence for the association between health expenditure and the health poverty vulnerability index. In other words, excessive health expenditures may lead households to fall into a poverty trap, as they may have to cut back on other necessities, cut back on other expenditures, or even incur debt to pay for healthcare, ultimately trapping them in a cycle of poverty.

### Health risk response capacity and health poverty vulnerability

#### The impact of household size on health poverty vulnerability

There was a positive association between family size and health poverty vulnerability, with a regression coefficient of 0.05 (see Table 5). This indicated that larger family size was associated with a higher probability of falling into health poverty vulnerability. This is likely because a larger family size usually means that more family members, including children and elderly individuals, require financial support. The more family members there are, the greater the health risk shock and the greater the financial burden borne, thereby leading to increased vulnerability to health poverty [25]. Owing to their limited household income, larger households may face more competition and resource allocation problems, including the allocation of medical resources, educational resources, etc., which may result in family members not being able to receive adequate medical attention and care, which, in turn, affects the health and well-being of the family [59].

#### The impact of household assets on health poverty vulnerability

Both household income and property were negatively associated with health poverty vulnerability, with regression coefficients of −0.06 and −0.07, respectively (see Table 5). Households with higher incomes or more property ownership have a lower probability of falling into health poverty because households with more assets have a greater ability to afford medical costs and other health expenditures. Conversely, households with lower household assets are often unable to afford the high cost of medical care and may have to opt for delayed access to medical care or low-quality medical services. There is also an interaction between household assets and health poverty vulnerability, with poverty and health problems often influencing each other in a vicious cycle. Households with no or few household assets are more vulnerable to poverty due to illness [35].

#### The impact of health insurance on health poverty vulnerability

There was a significant positive association between participation in basic medical insurance and health poverty vulnerability, with a regression coefficient of 0.58 (see Table 5). This finding is consistent with previous studies [11, 75], suggesting that the role of basic medical insurance in preventing health poverty vulnerability among low-income groups remains limited. A possible explanation is that the leverage effect of medical insurance may increase the utilization of healthcare services among low-income populations; however, even after reimbursement, out-of-pocket expenses remain substantial. In addition, limited medical resources, coupled with unfamiliarity with policy details and healthcare procedures, further constrain the effectiveness of medical insurance [11]. In the present study, low-income groups account for as much as 84% of the sample, which may have further amplified these challenges.

#### The impact of social capital on health poverty vulnerability

The availability of long-term care from relatives and friends was negatively associated with health poverty vulnerability, with a regression coefficient of −0.01 (see Table 5). Previous studies have shown that social capital helps families to reduce health poverty vulnerability given that relatives and friends can provide emotional support and life care assistance, thereby alleviating, to some degree, the economic and psychological burdens of the family and reducing vulnerability to health poverty [62]. However, the availability of financial support from relatives and friends was positively associated with health poverty vulnerability, with a regression coefficient of 0.01 (see Table 5), because those families who need financial support from relatives and friends exhibit high health poverty vulnerability due to low income and unstable jobs, while the support from relatives and friends proves to be inadequate.

### Health risk resilience and health poverty vulnerability

#### The impact of age on health poverty vulnerability

The results of the analysis indicate that age was positively associated with health poverty vulnerability, with a regression coefficient of 0.02 (see Table 5). Minimal health awareness, poor health status, a gradual decline in physical function, reduced work capacity and declining socioeconomic status are important reasons why rural elderly people return to poverty due to illness [14]. For those rural senior citizens who have no savings, when they are struck by illness, their labor capacity decreases, and their risk resilience is weakened. Since their finances come primarily from the support of their offspring, when the financial support provided by their offspring is insufficient to cope with major medical expenses, both they and their offspring are highly vulnerable to health poverty and may fall into poverty at the same time [37, 60].

#### The impact of level of education on health poverty vulnerability

The results of the analysis revealed that the highest educational level had a significant negative association with health poverty vulnerability, with a regression coefficient of −0.01 (see Table 5). Some studies have shown that high educational attainment reduces health poverty vulnerability [70] and that people with more years of education have a greater ability to acquire wealth and a greater awareness of health management [71]. This is because people with higher education usually have increased health knowledge and self-care awareness, and therefore, they are better able to understand the prevention and treatment of health problems and adopt positive health behaviors that lead to a lower risk of disease. Additionally, better educated individuals usually have better employment opportunities and higher socioeconomic status, making it relatively easy for them to obtain stable income and good medical insurance, which, in turn, results in their ability to better cope with the economic burdens caused by disease.

#### The impact of the labor force ratio on health poverty vulnerability

The research results revealed that the proportion of the labor force in the household was negatively associated with health poverty vulnerability, with a regression coefficient of −0.18 (see Table 5). This suggests that household with a higher proportion 0f working-age members tend to exhibit a lower health poverty vulnerability index and are associated with a reduced probability of falling into health-related poverty. A high proportion of the labor force in the household means that more family members are involved in labor, which is associated with higher total household income. Higher income, in turn, is linked to a better capacity to manage health-related expenditures such as medical and nutritional costs, and is correlated with a lower risk of economic poverty.

### Limitations

This study had several limitations. First, this study employed a convenience sampling method in only three prefecture-level cities in Jiangxi Province (*n* = 255), and the sample used for the study was not representative of rural households in central China. Therefore, research findings from this study cannot be generalized to rural households in central China or at the national level. In addition, because this was a cross-sectional study, no causal inference between independent and dependent variables could be drawn from the study. The present study can only reveal associational, rather than causal, relationships between three health risk related variables (health shocks, health risk response capacity, and health risk resilience) and health poverty vulnerability. Furthermore, due to limitations about our survey questionnaire, key potential confounders such as the presence of specific poverty alleviation program support, local healthcare infrastructure quality, and the distance to the nearest medical facility were not included in our regression model. This may lead to potential biases on regression results. Last but not least, the study cannot fully address the endogeneity problem of health shocks, which may weaken our research findings. The findings should be treated with caution.

## Conclusions

Based on risk theory, this study constructed a dynamic assessment framework for health-related poverty vulnerability, demonstrating that rural households in Jiangxi Province, China, has remained susceptible to returning to poverty due to health shocks. Our findings indicated that catastrophic health expenditures and labor loss were positively associated with increased vulnerability at the individual and household levels. In contrast, effective chronic disease management appeared to be correlated with reduced health vulnerability, possibly reflecting the supportive role of established social security mechanisms. Regarding health risk response capacity, asset accumulation and social capital may serve as important buffers, though their protective associations were context-dependent. Notably, enrollment in basic health insurance was linked to higher health-related poverty vulnerability, suggesting limited effectiveness in mitigating vulnerability among low-income groups. From a risk resilience perspective, higher educational attainment and a greater proportion of working-age family members were associated with lower vulnerability, though underlying mechanisms warranted further investigation. These results underscore the need for a resilient health security system that accurately identifies vulnerability pathways and integrates strengthened social protection, asset-building initiatives, and human capital investments to enhance the capacity of rural households to withstand health shocks.

## Data Availability

The datasets used and/or analyzed during the current study are available from the corresponding author on reasonable request.

## References

[1] Alwang J, Siegel PB, Jorgensen S, editors. Vulnerability: a view from different disciplines. Washington, D.C.: The World Bank. 2001.

[2] Banyuetan Network. Ganzhou city's case study summary report on poverty alleviation. 2021.

[3] Beck U. Risk society: towards a new modernity. London: SAGE Publications Ltd; 1992.

[4] Burt RS. Structural holes: the social structure of competition: Structural holes : the social structure of competition. 1995. 10.4159/9780674029095.

[5] Central Committee of the Communist Party of China, State Council. Decision of the central committee of the communist party of China and the state council on winning the battle against poverty. Beijing Review. 2015. http://www.beijingreview.com.cn/minsheng/201512/t20151208_800044364.html.

[6] Chaudhuri S, Jalan J, Suryahadi A. Assessing household vulnerability to poverty from cross-sectional data: a methodology and estimates from Indonesia. discussion papers. 2002. https://academiccommons.columbia.edu/doi/10.7916/D85149GF.

[7] Chen H. Health poverty and health equity. Academic Forum. 2010;33(07):1–6+68. https://link.cnki.net/doi/10.16524/j.45-1002.2010.07.016.

[8] Dercon S, Krishnan P. Vulnerability, seasonality and poverty in Ethiopia. Journal of Development Studies. 2000;36(6):25-53.

[9] Chen K, Qiu J, Wang W, Hu Q, Xu N, Qiao H. Identification and analysis of influencing factors of multidimensional health poverty in rural areas of Northwest China. Sci Rep. 2024;14(1):28952. 10.1038/s41598-024-80628-3. 39578607 10.1038/s41598-024-80628-3 PMC11584661

[10] Dong WH, Wu J, Yu CQ, Song XY, Lyu J, Guo Y, et al. Self-rated health measures and their relations to all-cause and cardiovascular mortality in adults from 10 regions of China. Chin J Epidemiol. 2021;42(5):763–70. 10.3760/cma.j.cn112338-20200622-00872. 10.3760/cma.j.cn112338-20200622-00872 34814465

[11] Du J, Guo W, Wang W, Chen K, Qiao H. Relationship between the health poverty vulnerability and multimorbidity patterns identified with latent class analysis aged 45 years or more adults in Northwestern China: A cross-section study. Medicine. 2024;103(1). 10.1097/MD.0000000000036746 10.1097/MD.0000000000036746 PMC10766289 38181282

[12] Du YZ, Lin PP. A review of poverty vulnerability research. Rural Econ Sci-Technol. 2021;32(12):184–6.

[13] Fang LM. Health risks and social determinants of health among middle-aged and elderly rural residents. Insurance Studies. 2017(05):117–27. https://link.cnki.net/doi/10.13497/j.cnki.is.2017.05.010.

[14] Fang YF, Zou W. Capability investment, health shocks, and poverty vulnerability. Dyn Econ. 2013;07:36–50.

[15] Feng X, Ding D. Did the healthy cities pilot policy improve household health poverty? An empirical study from China. Cities. 2025;167:106381. 10.1016/j.cities.2025.106381

[16] Fu FX. A study of the effect of social capitaland health risks on farmers' poverty vulnerability [Master's thesis]. Northwest A&F University. 2019.

[17] Gao S, Shi C, Tang J. The alleviation of household-level poverty vulnerability through ability enhancement and empowerment: A case study of Taihang mountain contiguous poverty-stricken Areas. China Rural Survey. 2020(01):61-75. 10.20074/j.cnki.11-3586/f.2020.01.005

[18] General Office of the National Health Commission. Notification on enhancing family doctor contractual services in 2019. 2019. https://www.nhc.gov.cn/jws/s7881/201904/4cb8d9c938fd4ec08cba94bd92f64fcf.shtml

[19] Genoni ME. Health shocks and consumption smoothing: evidence from Indonesia. Econ Dev Cult Change. 2012;60:475–506. 10.1086/664019.

[20] Granovetter MS. The strength of weak ties. Am J Sociol. 1973;78(6):1360–80. 10.1086/225469.

[21] Grossman M. On the concept of health capital and the demand for health. J Polit Econ. 1972;80(2):223–55. https://www.scribd.com/document/584818319/1972-Grossman-on-the-Concept-of-Health-Capital.

[22] Guo B, Xie X, Wu Q, Zhang X, Cheng H, Tao S, et al. Inequality in the health services utilization in rural and urban China: a horizontal inequality analysis. Medicine (Baltimore). 2020;99(2):e18625. 31914043 10.1097/MD.0000000000018625 PMC6959938

[23] Heistaro S, Jousilahti P, Lahelma E, Vartiainen E, Puska P. Self rated health and mortality: a long-term prospective study in eastern Finland. J Epidemiol Community Health. 2001;55(4):227–32. 10.1136/jech.55.4.227. 11238576 10.1136/jech.55.4.227 PMC1731868

[24] Holling CS. Resilience and stability of ecological systems. Annu Rev Ecol Evol Syst. 1973;4:1–23. 10.1146/annurev.es.04.110173.000245.

[25] Hu SY, Wang WL, Gao BK, Qiao H. Health poverty vulnerability and its determinants of rural residents in Ningxia. Rural Econ Sci-Technol. 2022;33(07):224–6+54.

[26] Jia HY, Wang JM. Theoretical logic, realistic challenges and path selection of mitigating relative poverty through health in rural areas. Yimeng Cadre Coll J. 2023(04):20–26. 10.13723/j.yxysh.2018.05.003.

[27] Jiangxi Provincial Bureau of Statistics, Jiangxi Province Seventh National Population Census Leading Group Office. Jiangxi province seventh national population census bulletin. Nanchang, Jiangxi Province. 2021. https://tjgb.hongheiku.com/11403.html.

[28] Kalla K. Understanding the concept of age: when determing the capabilities of leaders in post-modern organizations. 2006. https://warwick.ac.uk/fac/soc/wbs/conf/olkc/archive/olkc1/papers/328_kalla.pdf.

[29] Lai YQ, Li Y, Wu QH, Zhang XY, Sun H, Lian LB, et al. Establishment of an index system and multidimensional indexes forhealth poverty measurement: a literature review and Delphi methodstudy. Intro Public Health China. 2023;39(06):689–94. https://link.cnki.net/urlid/21.1234.R.20230421.1055.002.

[30] Liao L, Kong S, Du M. The effect of clean heating policy on individual health: Evidence from China. China Economic Review. 2025; 89(6501):102309. 10.1016/j.chieco.2024.102309

[31] Ligon E, Schechter L. Measuring Vulnerability. The Economic Journal. 2003;113(486):C95-C102. 10.1111/1468-0297.00117

[32] Li CS, Wang YY, Song MS, Qiao H. Analysis of the status quo and Influencing factors of health service utilization of ruralResidents in Ningxia from the perspective of health poverty vulnerability. Chinese General Practice. 2025:1-11. 10.12114/j.issn.1007-9572.2023.0475

[33] Li MK, Du XY, Liu YL. The current situation and recommendation of the medical assistance system in China. Chin Health Econ. 2024;43(06):50–3.

[34] Li X, Shen JJ, Lu J, Wang Y, Sun M, Li C, et al. Household catastrophic medical expenses in eastern China: determinants and policy implications. BMC Health Serv Res. 2013;13:506. 24308317 10.1186/1472-6963-13-506 PMC4234144

[35] Lin N. From individual to society: a perspective of social capital. Soc Sci Front. 2020;02:213–23.

[36] Liu F, Zhu XP, Yin XB, Wu Q, Li XY, Xie CY, et al. Progress on family caregiver′s burden of the elderly patients. Chin J Pract Nurs. 2018;34(10):796–800. 10.3760/cma.j.issn.1672-7088.2018.10.017.

[37] Liu W, Zhu YC. The effecets of the health risks on vulnerability to poverty of farmers. Hubei Agric Sci. 2014;53(13):3216–20. https://link.cnki.net/doi/10.14088/j.cnki.issn0439-8114.2014.13.123.

[38] Liu Y, Liu HM, Li AC, Li G, Guan Z, Wang J. Analysis on connotation of health poverty and its vulnerability. Chin J Public Health Manag. 2019;36(5):363–365, 82.

[39] Luo ZY. Study on Influencing factors of healthpoverty among the elderly in rural areasfrom the perspective of health risk theor [Master’s thesis]. Wuhan University of Science and Technology. 2023. https://link.cnki.net/doi/10.27380/d.cnki.gwkju.2023.000317.

[40] Ma YL, Xiang Q, Yan C, Liao H, Wang J. Poverty vulnerability and health risk action path of families of rural elderly with chronic diseases: empirical analysis of 1,852 families in Central and Western China. Front Public Health. 2022(10). 10.3389/fpubh.2022.776901 PMC8882595 35237547

[41] Malmberg Calvo C, Das Gupta M, Grootaert CN, Kanbur R, Kwakwa V, Lustig NC. World development report 2000/2001: attacking poverty - overview. World development indicators Washington, DC. 2001. http://documents.worldbank.org/curated/en/673161468161371338.

[42] National Bureau of Statistics of the People's Republic of China. Statistical Bulletin on the National Economic and Social Development of the People's Republic of China for 2015. 2016. https://www.stats.gov.cn/sj/zxfb/202302/t20230203_1899041.html.

[43] Oliveira GM, Vidal DG, Ferraz MP, Cabeda JM, Pontes M, Maia RL, et al. Measuring health vulnerability: an interdisciplinary indicator applied to mainland Portugal. Int J Environ Res Public Health. 2019. 10.3390/ijerph16214121. 31731572 10.3390/ijerph16214121 PMC6862183

[44] Pham A, Mukhopadhaya P. Estimating poverty and vulnerability to monetary and non-monetary poverty: the case of Vietnam. Soc Indic Res. 2022;159(1):281–317. 10.1007/s00181-020-01991-4. 34253938 10.1007/s11205-021-02747-y PMC8264820

[45] Qian L, Wang H. Vulnerability measurement and influencing factors analysis of relative poverty in rural families. Agric Econ Manag. 2022;02:49–58.

[46] Qiao JF, Guo MY. Can basic public services effectively improve the quality of poverty alleviation? —— based on the perspective of multidimensional poverty and the vulnerability of multidimensional poverty. Public Finance Res. 2021(12):48–62. https://link.cnki.net/doi/10.19477/j.cnki.11-1077/f.2021.12.007.

[47] Qiao SD. Risk society and political ethics. J Shanghai Jiaotong Univ (Philosophy and Social Sciences). 2011;19(04):12–18. 10.13806/j.cnki.issn1008-7095.2011.04.007.

[48] Ross CE, Mirowsky J. Does medical insurance contribute to socioeconomic differentials in health? Milbank Q. 2000;78(2):291–152. 10.1111/1468-0009.00171. 10934995 10.1111/1468-0009.00171 PMC2751153

[49] Sen A. Development as freedom (1st. ed). New York: Knopf. 1999(16):366. http://www.loc.gov/catdir/bios/random051/99031061.html.

[50] Spitzer S, Weber D. Reporting biases in self-assessed physical and cognitive health status of older Europeans. PLOS ONE. 2019;14. 10.1371/journal.pone.0223526 PMC6783110 31593576

[51] Sun SM. Analysis of the impact of social capital on the health poverty vulnerability of rural residents in the post-poverty era [Master’s thesis]. Nanjing University of Chinese Medicine. 2021. https://link.cnki.net/doi/10.27253/d.cnki.gnjzu.2021.000691.

[52] The State Council Information Office of the People's Republic of China. Poverty alleviation: China's experience and contribution. Beijing: Xinhua News Agency; 2021.https://www.gov.cn/zhengce/2021-04/06/content_5600000.htm.

[53] Townsend P. Poverty in the United Kingdom: a survey of household resources and standards of living. Berkeley: University of California Press; 1979. p. 12–6. 10.1525/9780520325760.

[54] Tu BQ, Li HJ, Tang H. Health shock, cocial capital and household economic vulnerability in rural areas. South China J Econ. 2018(12):17–39. https://link.cnki.net/doi/10.19592/j.cnki.scje.360389.

[55] Wade RH. Making the world development report 2000: attacking poverty. World Dev. 2001. 10.1016/S0305-750X(01)00047-X.

[56] Wagstaff A, Lindelöw M, World Bank. Can insurance increase financial risk? the curious case of health insurance in China. Washington, D.C.: World Bank; 2005. 10.1016/j.jhealeco.2008.02.002 18342963

[57] Wagstaff A. The economic consequences of health shocks: evidence from Vietnam. J Health Econ. 2007;26(1):82–100. 10.1016/j.jhealeco.2006.07.001. 16905205 10.1016/j.jhealeco.2006.07.001

[58] Wang DH. Health human capital, economic growth and poverty trap. Econ Res. 2012;47(06):143–55.

[59] Wang HY, Du T. Exploration on the mode and path of health poverty governance under the perspective of risktheory: a case study of “Three-Districts” project in Shanxi Province. Chin J Health Policy. 2021;14(01):2–9.

[60] Wang JY. Analysis on contributing factors of long-term care service demand in urban and rural areas. China Med Insurance. 2022(05):50–6. https://link.cnki.net/doi/10.19546/j.issn.1674-3830.2022.5.010.

[61] Wang W, Chen K, Xiao W, Du J, Qiao H. Determinants of health poverty vulnerability in rural areas of Western China in the post-poverty relief era: an analysis based on the Anderson behavioral model. BMC Public Health. 2024;24(1):459. 38355428 10.1186/s12889-024-18035-6 PMC10865669

[62] Wei Y, Tang BM. Health shock, social capital and rural household poverty vulnerability. J Stat Inform. 2022;37(10):103–16.

[63] World Bank. World development report 2000/2001: attacking poverty. The World Bank. 2000.

[64] World Bank. Poverty and shared prosperity 2020: reversals of fortune. Washington, DC. 2020. https://www.worldbank.org/en/publication/poverty-and-shared-prosperity.

[65] World Health Organization, World Bank. Tracking universal health coverage: 2023 global monitoring report. Geneva; 2023.https://www.who.int/data/gho/data/themes/topics/servicecoverage.

[66] Xie E. Catastrophic out-of-pocket payments for health and poverty: micro evidence from China. J Shanghai Univ Finance Econ. 2023;25(3):47–63, 107.

[67] Xie JZ, Wang WT, Che SF. Evaluation of social resilience and analysis in distribution characteristics. J Hunan Univ (Social Sciences). 2016;30(03):85–93. https://link.cnki.net/doi/10.16339/j.cnki.hdxbskb.2016.03.014.

[68] Xu K, Evans DB, Kawabata K, Zeramdini R, Klavus J, Murray CJ. Household catastrophic health expenditure: a multicountry analysis. Lancet. 2003;362(9378):111–7. 10.1016/s0140-6736(03)13861-5. 12867110 10.1016/S0140-6736(03)13861-5

[69] Yan BL. Analysis on the measurement and influencing factors of health and poverty vulnerability of patients with chronic diseases-taking the three cities of northern Jiangsu as an example [Master’s thesis]. Nanjing University of Chinese Medicine. 2019.

[70] Yang B, Xie ZG. Constructing the proactive poverty alleviation theory by drawing on the principle of comprehensive risk management. Shanghai Insurance. 2019;04:14–8.

[71] Yang L, Wang SG. Multidimensional poverty measurement and decomposition of households in poor areas—based on the China rural poverty monitoring household survey in 2010. Popul J. 2015;37(02):15–25.

[72] Yang YF. Since the 18th National Congress of the Communist Party of China, significant achievements have been made in health poverty alleviation—basic medical care for rural poor population has been fully guaranteed. People's Daily. 2022.

[73] Yang YZ, Shangguan SY, Zhen XD, Fang XM. The health effects of geographic accessibility of rural medical services from the perspective of equity. Chin Rural Econ. 2024(11):102–124. 10.20077/j.cnki.11-1262/f.2024.11.005.

[74] Yin SJ. Research on the long-term care needs of the elderly in China [Doctoral's thesis]. Renmin University of China. 2011.

[75] Yuan X, Yang X, Zhou X. The poverty prevention effects of health insurance: evidence from China's basic medical insurance program. Frontiers in Public Health. 2025. 10.3389/fpubh.2025.1576146 PMC12058691 40342506

[76] Yu X. Research on the impact of health risk factors on poverty vulnerability ofmiffle-aged and elderly people [Master’s thesis]. Chongqing Medical University. 2022. 10.27674/d.cnki.gcyku.2022.000883.

[77] Zhang YQ. Social capital and the health vulnerability of poor farmers: inhibition or promotion? —— based on the data from the China household tracking survey. Xinjiang State Farms Economy. 2022(06):1–9. https://link.cnki.net/urlid/65.1048.F.20220506.0942.002.

[78] Zhang Y, Wan GH. (Eds.). Can we predict vulnerability to poverty. 2008. https://hdl.handle.net/10419/45164.

[79] Zhao X, Wang X, Ren Z, Zhang C. The impact of health shocks on poverty vulnerability: evidence from rural households in China. Front Public Health. 2025;13:1657269. 10.3389/fpubh.2025.1657269 10.3389/fpubh.2025.1657269 PMC12426276 40951400

[80] Zheng L., Peng L. Effect of major illness insurance on vulnerability to poverty: evidence from China. Front Public Health. 2021(9). 10.3389/fpubh.2021.791817 PMC8733306 35004594

[81] Zuo T, Xu XY. The “poverty-disease” vicious cycle in rural areas and the construction of chain health security system in precision poverty alleviation. J Southwest Minzu Univ (Humanities and Social Sciences Edition). 2017;38(01):1–8.

